# Changes in soil carbon sequestration and emission in different succession stages of biological soil crusts in a sand-binding area

**DOI:** 10.1186/s13021-021-00190-7

**Published:** 2021-09-13

**Authors:** Bo Wang, Jing Liu, Xin Zhang, Chenglong Wang

**Affiliations:** 1 College of Desert Control Science and Engineering, Inner Mongolia Agricultural University, Hohhot, 010018 China; 2 Inner Mongolia Academy of Forestry Sciences, Hohhot, 010010 China; 3 Institute of Water Resource for Pasturing Area, Ministry of Water Resources, Hohhot, 010019 China

**Keywords:** Hobq Desert, Carbon emission, Soil carbon density, Biological soil crusts, Hydrothermal factors

## Abstract

**Background:**

We investigated the spatio-temporal dynamics of soil carbon dioxide (CO_2_)- and soil methane (CH_4_)-flux during biological soil crust (BSCs) deposition in a sand-binding area in the eastern Chinese Hobq Desert. The trends in soil organic carbon (C) content and density were analyzed during this process. The sampling sites comprised a mobile dune (control) and those with algal, lichen, and moss crust-fixed sands. The desert soil CO_2_- and CH_4_-flux, temperature, and water content were measured from May to October in 2017 and 2018. Simultaneously, organic C content and density were measured and analyzed by stratification.

**Results:**

The spatio-temporal variation in desert soil CO_2_-flux was apparent. The average CO_2_- fluxes in the control, algal, lichen, and moss sites were 1.67, 2.61, 5.83, and 6.84 mmol m^−2^ h^−1^, respectively, during the growing season, and the average CH_4_-fluxes in the four sites were − 1.13, − 1.67, − 3.66, and − 3.77 µmol m^−2^ h^−1^, respectively. Soil temperature was significantly positively correlated with CO_2_-flux but could not influence CH_4_ absorption, and C flux had minimal correlation with soil water content. The soil total organic C density at all sites was significantly different and decreased as follows: moss > lichen > algal > control; moreover, it decreased with soil depth at all sites. The accumulation of desert soil organic C could enhance soil C emissions.

**Conclusion:**

In a semi-arid desert, artificial planting could promote sand fixation and BSCs succession; therefore, increasing the C storage capacity of desert soils and decreasing soil C emissions could alter the C cycle pattern in desert ecosystems. Soil temperature is the major factor controlling desert soil CO_2_ flux and vegetation restoration, and BSCs development could alter the response patterns of C emissions to moisture conditions in desert soils. The results provide a scientific basis for studying the C cycle in desert ecosystems.

## Background

Ecosystem carbon (C) stocks result from long-term C accumulation and comprise plant, litter, and soil C stocks. Their amounts can vary depending on ecosystem type, regional environmental conditions, and anthropogenic interventions, which are the theoretical basis for the enhancement of ecosystem C stocks through land-use/cover change. Soils are the largest C pools in terrestrial ecosystems, with a reservoir size approximately twice that of atmospheric C pools and thrice that of vegetation C pools [1]. Soil C content and density directly affect the net primary productivity of plants and are important indicators of soil fertility. The variation in ecosystem CO_2_ and CH_4_ emissions significantly affects C pools and is a direct or indirect contributor to global warming. Therefore, scientific questions about the stability of C pools and their distribution among different compartments, as well as the biogeochemical cycling of CO_2_ and CH_4_, have been at the heart of climate change research.

Soil C flux refers to the CO_2_ and CH_4_ flux at the soil surface, which is the main source of C emissions from the soil to the atmosphere [2]. In contrast, soil organic C (SOC) indicates the balance between the input of organic matter, such as biological residues, into the soil and its loss from the soil, mainly due to soil microorganism decomposition and soil respiration, which is an indicator for the direct measurement and evaluation of soil C storage capacity [3]. Therefore, small changes in soil C flux and organic C content, which are the important components of the pathway between the input and output of soil C pools, directly alter the C stocks in the pedosphere and atmosphere, thereby affecting ecosystem C cycling processes and global C balance [4]. The study of soil C fluxes and stocks in terrestrial ecosystems, especially the exploration of changes in soil C pools under different land-use patterns in the context of global climate change, can provide scientific basis for ecological management, such as plantation forest construction, natural forest protection, returning farmland to forests and grasslands, and desertification control, thereby clarifying its value and ensuring its rationality.

At present, extensive research has been conducted on soil C dynamics in natural ecosystems (e.g., forests, grasslands, and wetlands) and artificial ecosystems (e.g., urban green spaces and farmlands), mainly focusing on soil respiration rate, ecological stoichiometric characteristics, C and nitrogen distribution patterns, and C mineralization and turnover characteristics [5–8]. As an important component of terrestrial ecosystems, desert ecosystems are characterized by homogeneous vegetation, low coverage, and severe erosion. Desert soils account for approximately 9.5% of the total C stock in the pedosphere and play a crucial role in the global C cycle [9]. Therefore, it is of great significance to study the dynamic characteristics of desert soil C, especially soil surface C flux and underground C stocks, to accurately assess the C budget in desert areas and to formulate scientific and rational measures for their management and utilization.

In China, extensive desertification control has been carried out on desertified lands in arid and semi-arid regions as well as on the edges of large deserts and oasis extensions. The promotion of sand fixation by artificial planting can facilitate vegetation restoration and improve regional microhabitats, thus forming stable ecosystems with the natural succession of communities [10]. In this process, as the vegetation coverage increases, the vegetation-soil feedback affects soil physicochemical properties, root distribution, microbial colonization, and soil fauna activity, while also promoting the development of biological soil crusts (BSCs) and a tendency toward mature succession [11]. Algae crust is the early developmental stage of BSCs, and an increase in algal and fungal biomass is a prerequisite for the formation of lichen and algae crusts; lichen crust continuously improves soil texture and provides a stable topsoil environment for the survival of moss. Current research on BSCs is mainly concerned with its effects on the spatial distribution of soil moisture, soil microbial community, and vegetation structure [12–14]. Additionally, BSCs have been shown to be an essential C source that can change soil respiration characteristics in desert ecosystems [15]. However, there is still a lack of systematic studies on the changes in soil C fluxes and stocks during the succession of BSCs and their mechanisms of influence. The Hobq Desert is the seventh largest desert in China, occupying the narrow strip of land between the northern part of the Ordos Plateau and the south bank of the Yellow River. It is one of the major sand sources in northern China. In this study, we examined the soil of an artificial sand-fixing area in the eastern Hobq Desert, using BSCs development as the basis for the division of sample sites, which were as follows: mobile dunes without crusts, algal crusted sandy land in the early developmental stage, lichen crusted sandy land in the middle stage, and moss crusted sandy land in the mature stage, with the aim of clarifying: (1) the spatio-temporal dynamics of soil CO_2_ and CH_4_ flux and its environmental controlling factors, (2) the dynamic variations in SOC content and density, and (3) the synergistic relationship between C flux and C stock in desert soils during vegetation restoration and BSCs succession.

## Results

### Variations of CO_2_ fluxes and hydrothermal factors in desert soil

**Fig. 1:**
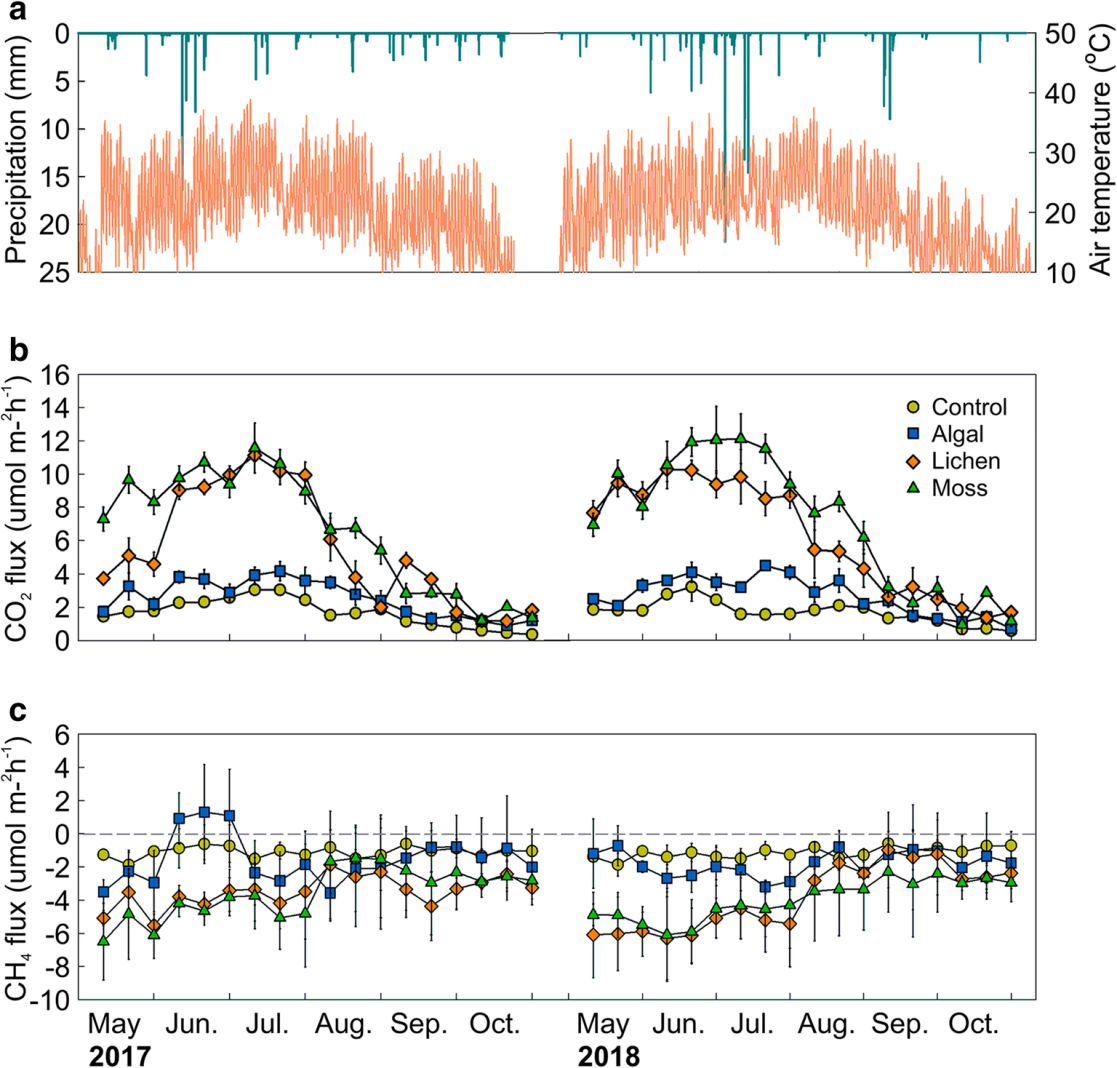
Time series of soil CO_2_ and CH_4_ fluxes and climatic factors. Time series of climatic factors, and soil CO_2_ and CH_4_ fluxes at Hobq Desert (Inner Mongolia, China) in 2017 and 2018. The top panel shows precipitation and air temperature in the study area, the bottom two panels show soil CO_2_ and CH_4_ fluxes at the control, algal, lichen, and moss sites. Data are shown as means with standard errors

The climate of the study area was characterized by the clear concurrence of precipitation and high temperatures, with more rainfall and higher temperatures during the growing season. During the growing seasons of 2017 and 2018, the cumulative precipitation amounts were 220.2 and 284.4 mm, respectively, and the average temperatures were 20.8 and 19.9 °C, respectively (Fig. 1).

There were no significant differences in soil temperature among the control, algal, lichen, and moss sample sites (*P* > 0.05), except in June. Seasonal variations showed distinct unimodal curves (Table 1), with relatively high soil temperatures in June and July, and the lowest temperature occurring in late October. Certain differences were observed in the soil water content of the four sample sites, whereby the soil water content of the control site was significantly higher than that of the algal and lichen sites and slightly higher than that of the moss site. All four sample sites showed evident seasonal variation in their soil water content, which was lower in June and July, and higher in May and October, thus exhibiting a dynamic pattern that was opposite to that of soil temperature.

**Table 1:** Mean soil temperature and water content at different soil depths during developmental stages of BSC

Sample plots	Item	May	June	July	August	September	October	Growing season
Control	Soil water content (%)	9.24 ± 1.31^Ab^	7.63 ± 0.93^Ca^	6.66 ± 0.43^ Da^	7.61 ± 0.61^Ca^	9.42 ± 1.27 ^Aa^	8.45 ± 0.23^Bb^	8.16 ± 0.82^a^
Algal		8.75 ± 0.79^Bc^	6.41 ± 0.67^Cc^	4.28 ± 1.73^Ec^	5.32 ± 1.89^Dc^	9.83 ± 1.03^Aa^	8.18 ± 1.83^Bb^	7.22 ± 1.34^b^
Lichen		9.11 ± 1.15^Abc^	7.15 ± 0.89^Bb^	5.56 ± 1.22^Cb^	5.75 ± 1.76^Cbc^	9.39 ± 0.94^Aa^	7.85 ± 1.69^Bb^	7.58 ± 1.28^ab^
Moss		10.21 ± 1.04^Aa^	6.46 ± 1.78^Bc^	5.05 ± 1.94^Cbc^	6.14 ± 2.29^Bb^	9.91 ± 1.19^Aa^	9.65 ± 2.13^Aa^	7.93 ± 1.73^a^
Control	Soil temperature (°C)	22.51 ± 0.35^ Da^	35.64 ± 0.54^Aa^	30.72 ± 0.53^Bb^	27.16 ± 1.40^Ca^	21.74 ± 0.57^Db^	14.14 ± 0.37^Ea^	25.31 ± 0.69^a^
Algal		18.92 ± 0.99^Cc^	28.01 ± 0.38^Bd^	32.64 ± 0.76^Aa^	27.85 ± 0.69^Ba^	20.40 ± 0.37^Cb^	12.72 ± 0.22^Db^	23.42 ± 0.76^b^
Lichen		20.85 ± 1.20^Cb^	33.49 ± 0.27^Ab^	32.45 ± 1.26^Aa^	27.73 ± 0.49^Ba^	20.78 ± 0.50^Cb^	12.82 ± 0.42^Db^	24.62 ± 0.79^a^
Moss		20.47 ± 1.03^Db^	29.30 ± 1.17^Bc^	32.82 ± 1.34^Aa^	27.91 ± 0.60^Ba^	25.01 ± 1.17^Ca^	13.17 ± 1.66^Eb^	24.78 ± 1.21^a^

The lower case letters indicate significant differences at 0.05 level among the four sites in the same month; the uppercase letters indicate significant differences at 0.05 level during the 6 months at the same site

*BSCs* biological soil crusts

During the growing season, clear spatio-temporal variations were observed in desert soil CO_2_ and CH_4_ fluxes, with significant differences in C emissions at different stages of vegetation recovery and BSCs development and in different seasons (*P* < 0.05). Furthermore, the dynamic CO_2_ flux patterns were consistent with soil temperature, exhibiting unimodal curves (Fig. 1). The average CO_2_ fluxes in the control, algal, lichen, and moss sites were 1.67, 2.61, 5.83, and 6.84 mmol m^−2^ h^−1^, respectively, during the growing season, and the average CH_4_ fluxes for the four sites were − 1.13, − 1.67, − 3.66, and − 3.77 µmol m^−2^ h^−1^, respectively. The maximum CO_2_ flux in the control site was observed in early July, that for the algal site in late July, and those for the lichen and moss sites in early June. The minimum CO_2_ flux and CH_4_ absorption values for all four sample sites were observed in late October.

A two-way analysis of variance (ANOVA) (Table 2) showed that the effects of BSCs succession, sampling time, and their interaction on desert soil CO_2_ and CH_4_ fluxes were all highly significant (P < 0.01), while the effects of BSC succession stages on soil CO_2_ and CH_4_ fluxes were greater than those of the sampling time. The soil temperature and water content were significantly affected by only sampling time (P < 0.01) and not by BSCs succession stages or their interaction. This suggests that vegetation restoration and BSCs development could alter the C flux patterns of desert soils but had little effect on soil hydrothermal redistribution.

**Table 2:** Effect of succession stages of biological soil crusts and sampling time on different soil parameters

Item	Source of variation	*df*	Mean square	F-value	*P*-value	Partial η^2^
Soil CO_2_ fluxes	Succession stages	3	125.427	78.480	< 0.001**	0.797
	Time	2	134.991	84.464	< 0.001**	0.738
	Succession stages × time	6	20.057	12.550	0.004**	0.557
Soil CH_4_ fluxes	Succession stages	3	36.532	76.997	< 0.001**	0.794
	Time	2	21.652	45.634	< 0.001**	0.603
	Succession stages × Time	6	4.717	9.941	< 0.001**	0.499
Soil temperature	Succession stages	3	18.722	0.746	0.529	0.036
	Time	2	1138.474	45.389	< 0.001**	0.602
	Succession stages × time	6	19.087	0.761	0.603	0.071
Soil water content	Succession stages	3	2.994	1.152	0.335	0.054
	Time	2	53.195	20.477	< 0.001**	0.406
	Succession stages × time	6	1.191	0.459	0.836	0.044

The effect of succession stages of BSCs, sampling time, and their interaction on soil CO_2_ and CH_4_ fluxes, temperature, and water content

*indicates significant correlation at *P* < 0.05, and **indicates extremely significant correlation at *P* < 0.01

### Effects of hydrothermal factors on CO_2_ and CH_4_ fluxes

**Fig. 2:**
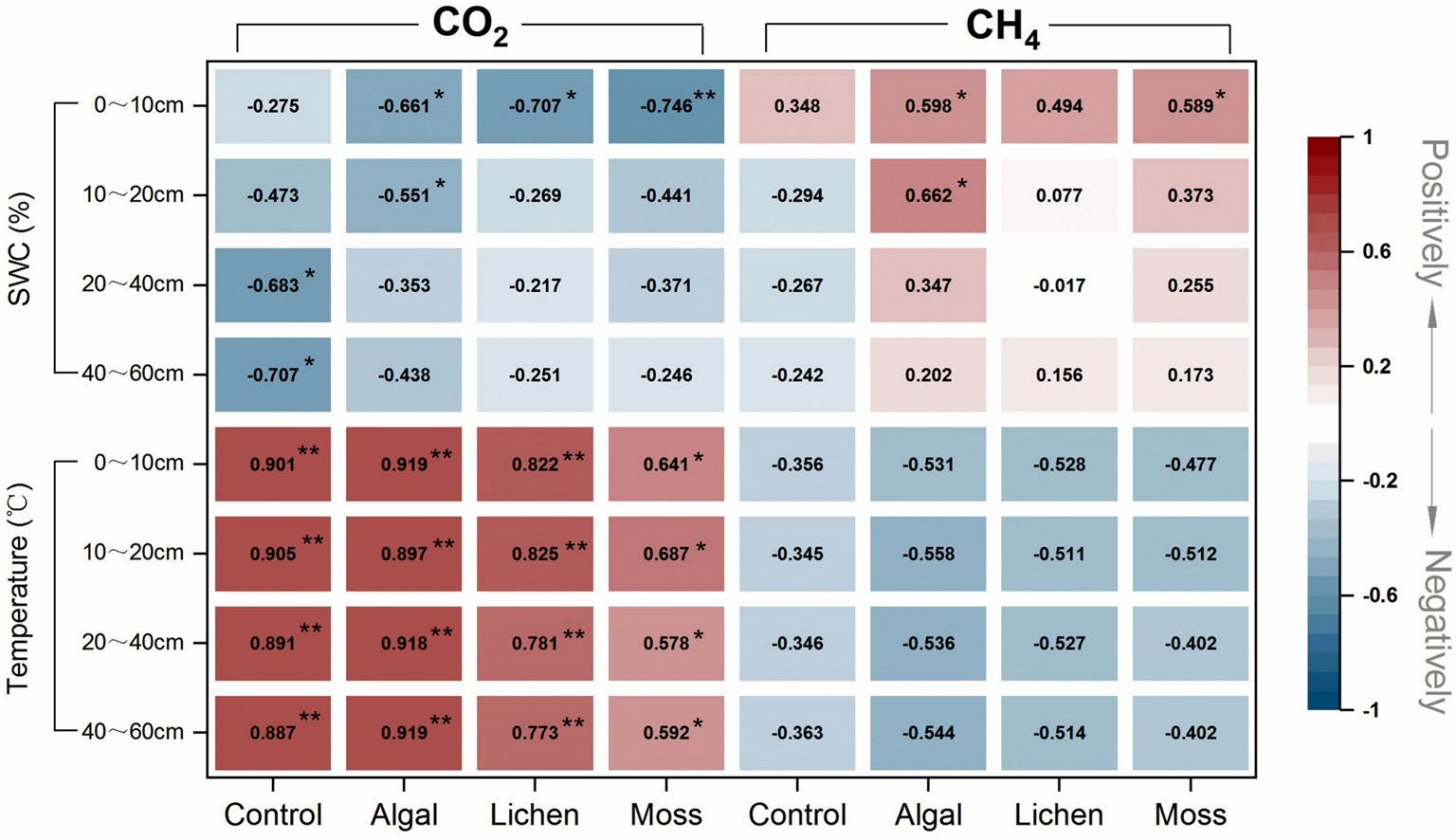
Results of Pearson’s correlation. Pearson’s correlation coefficients between desert soil CO_2_ and CH_4_ fluxes and soil hydrothermal factors for different sites. Positive correlations are indicated in orange and negative correlations in brown

The correlation analysis (Fig. 2) showed that the soil CO_2_ fluxes of the four sites were all positively correlated with soil temperature (*P* < 0.05); the nonlinear model reflects the effect of temperature at different sites as follows: control (*F* = 2.391 T^0.574^, *R*^2^ = 0.869), algal (*F* = 1.454 T^0.589^, *R*^2^ = 0.831), lichen (*F* = 12.445 ln (*T*) − 37.597, *R*^2^ = 0.763), and moss (*F* = 9.594 ln (*T*) − 25.083, *R*^2^ = 0.517). The effect of soil water content on CO_2_ fluxes varied across different sites. With the development of BSCs, the influence of shallow soil water content on CO_2_ fluxes gradually increased. CH_4_ fluxes were not correlated with soil temperature at all sites and were positively correlated with soil water content only in the surface layer in algal and moss sites. The results indicate that soil temperature is the major factor controlling desert soil CO_2_ flux, although it does not influence CH_4_ absorption. In addition, vegetation restoration and BSCs development could alter the response patterns of C emissions to moisture conditions in desert soils.

### Variations of organic carbon density in desert soil

**Fig. 3:**
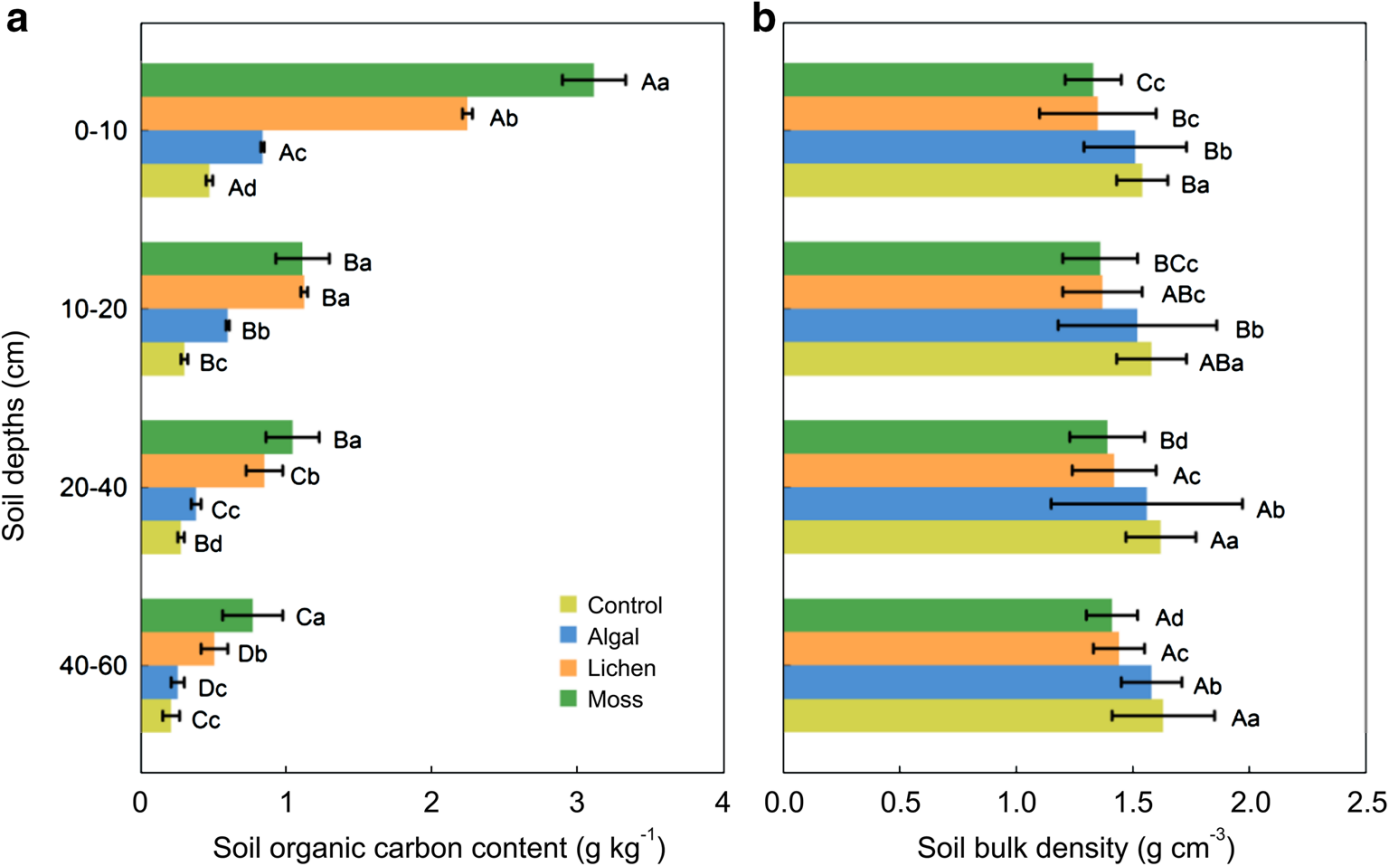
Soil organic carbon (SOC) content and bulk density variation in sampling sites. Variation of soil organic carbon (SOC) content and bulk density at different sites. Different capital letters indicate significant differences at 0.05 level among different soil depths at the same site; the different small letters indicate significant differences at 0.05 level among the four sites at the same soil depth

SOC content and bulk density varied significantly among different succession stages of BSCs and soil depths (*P* < 0.05). In the 0–60 cm layer, SOC content gradually increased with BSCs succession and decreased with soil depth (Fig. 3). The range of SOC content for the control, algal, lichen, and moss sites was 0.18–0.41, 0.22–0.73, 0.44–1.96, and 0.67–2.72 g kg^−1^, respectively. Among all sites, the moss site had the highest SOC content, which was 4.89, 2.93, and 1.28 times that of the control, algal, and lichen sites, respectively. Among the soil layers, the SOC content was the highest in the 0–10 cm soil layer, which was 3.85, 2.62, and 2.12 times that in the 10–20, 20–40, and 40–60 cm soil layers, respectively. The spatial variation of soil bulk density was the opposite of SOC, gradually decreasing with BSCs succession and increasing with soil depth.

**Fig. 4:**
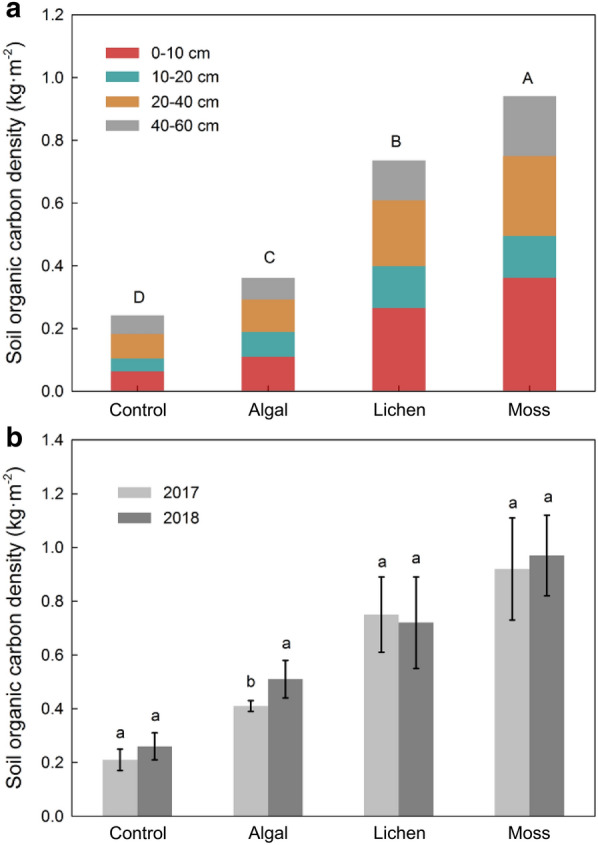
Characteristics of soil organic carbon density. Characteristics of soil organic carbon (SOC) density in the control, algal, lichen, and moss sites. The top panel shows the variation characteristics of SOC density with soil depth, different capital letters indicate significant differences between plots. The bottom panel shows annual variation of SOC density, different small letters indicate significant differences from year to year. Data are shown as means with standard errors

The total organic C (TOC) densities of desert soils at different stages of BSCs succession differed significantly (*P* < 0.05), which were 0.24, 0.36, 0.74, and 0.94 kg m^−2^ for the control, algal, lichen, and moss sites, respectively, thus showing a significant increasing trend with BSCs succession (Fig. 4). With the exception of control, the TOC density of surface soil (0–10 cm) accounted for the largest proportion of the soil profile in the other three sample sites, showing clear nutrient enrichment. The total SOC densities of the control, algal, and moss sites were all slightly higher in 2018 than in 2017, but the opposite was true for the lichen site.

The effects of BSCs succession and soil depth on SOC content, bulk density, and SOC density were all highly significant (*P* < 0.01), but their interaction only had significant effects on SOC content (Table 3). Moreover, the effect of BSCs succession on SOC density was greater than that of soil depth, indicating that vegetation restoration and BSCs development can significantly alter soil C stocks and promote soil C storage.

**Table 3:** Effects of succession stages on soil organic carbon (SOC) sequestration

Item	Source of variation	*Df*	Mean square	F-value	*P*-value	Partial η^2^
Soil organic carbon content	Succession stages	3	2.144	22.705	< 0.001**	0.831
	Soil depths	3	2.084	22.062	< 0.001**	0.752
	Succession stages × soil depths	9	0.306	3.240	0.019**	0.714
Soil bulk density	Succession stages	3	0.095	2023.000	< 0.001**	0.795
	Soil depths	3	0.014	292.867	< 0.001**	0.774
	Succession stages × soil depths	9	< 0.001	2.259	0.074	0.238
Soil organic carbon density	Succession stages	3	0.063	16.349	< 0.001**	0.726
	Soil depths	3	0.024	6.345	0.005**	0.633
	Succession stages × soil depths	9	0.006	1.618	0.192	0.089

The effect of different succession stages of biological soil crusts (BSCs), soil depths, and their interaction on soil organic carbon SOC sequestration

*indicates significant correlation at *P* < 0.05, and **indicates extremely significant correlation at *P* < 0.01

### Correlation between annual soil surface carbon fluxes and organic carbon density during the growing season

During the growing season, desert soil surface C fluxes gradually increased with vegetation recovery and BSCs succession. The annual soil surface C fluxes for the control, algal, lichen, and moss sites were 316.50, 492.04, 1102.81, and 1292.78 gC m^−2^ year^−1^, respectively. The Pearson’s correlation analysis (Table 4) showed that the annual surface C emission of desert soils undergoing vegetation recovery were significantly correlated with SOC density in the 0–10 cm and 10–20 cm layers (*P* < 0.05) as well as with that of the soil profile.

**Table 4:** Pearson’s correlation coefficients between desert soil surface C emissions and SOC density

Item	Soil carbon emissions	SOC density				
		0–10 cm	10–20 cm	20–40 cm	40–60 cm	0–60 cm
Soil carbon emissions	1	0.951*	0.966*	0.845	0.813	0.911*
Soil organic carbon density						
0–10 cm		1	0.835	0.997**	0.986*	0.892*
10–20 cm			1	0.848	0.866	0.905*
20–40 cm				1	0.974*	0.932*
40–60 cm					1	0.816
0–60 cm						1

*indicates significant correlation at *P* < 0.05, **indicates extremely significant correlation at *P* < 0.01

## Discussion

### Seasonal variation of desert soil CO_2_ and CH_4_ fluxes and response to hydrothermal factors

This study showed that clear seasonal variations could be observed in desert soil CO_2_ fluxes from the control, algal, lichen, and moss sites, all of which showed unimodal curves consistent with the soil temperature; however, there were no significant seasonal variation in CH_4_ flux, and the desert soils in all sites exhibited CH_4_ absorption. Furthermore, the correlation analysis revealed a significant positive correlation only between CO_2_ fluxes and soil temperature, indicating that soil temperature is the main factor controlling CO_2_ fluxes in desert soils, although change in soil temperature could not affect CH_4_ absorption. Liu et al. also found in a study on *Artemisia ordosica* shrubland in the Mu Us Desert that soil heterotrophic and autotrophic respiration were mainly controlled by soil temperature [16]. Furthermore, other researchers drew the same conclusion in studies on semi-arid desert grasslands [17] and an arid desert in northwest China [18]. The direct effect of soil temperature on soil CO_2_ fluxes primarily stems from the sensitivity of the components of C fluxes to temperature changes. The soil CO_2_ fluxes measured in this study were in the form of total soil respiration, which included plant root and rhizosphere microbial respiration, soil microbial respiration, soil animal respiration, BSCs respiration, and mycorrhizal respiration. Soil temperature can change the community composition structure of soil microorganisms as well as the number of microbial communities, and an increase in temperature within a certain range can promote microbial proliferation [19]. Soil temperature can also significantly affect microbial activity, and as temperature increases, an increasing number of molecules reach or exceed their own activation energy, thus accelerating the reaction and increasing the CO_2_ efflux [20]. In addition, the existing root biomass of plants is extremely sensitive to soil temperature changes [21], while living roots can perform autotrophic respiration and dead roots are substrates for heterotrophic respiration. Therefore, an increase in root biomass accumulation with increasing soil temperature will inevitably lead to an increase in soil CO_2_ flux. The insensitivity of CH_4_ absorption to soil temperature was mainly due to the fact that methane-oxidizing bacteria are often mesophilic, relatively insensitive to temperature changes and able to maintain high activity over a wide range of temperature change [22].

The effect of soil water content on soil CO_2_ and CH_4_ fluxes in arid and semi-arid desert areas is relatively complex. In this study, the soil water content had a weak effect on soil CO_2_ and CH_4_ fluxes. During the growing season, the soil water content only showed negative correlations with soil CO_2_ fluxes and positive correlations with CH_4_ fluxes in the surface layer of the BSC fixed sands, which is consistent with the results of studies on soil C fluxes in desert *Populus* plantations [23] and *Halostachys caspica* communities [24]. This could be attributed to the fact that in desert areas where water is scarce, the plant canopy begins to experience water stress when soil water content is low, and the proportion of soluble carbohydrates allocated to the roots will increase, thus resulting in higher root respiration and increased soil CO_2_ efflux [25]. Contrary to the results of the present study, researchers have reported a significant positive correlation between soil C flux and water content in arid sand burial areas [26] and desert *Haloxylon ammodendron* forests [27], whereas Li et al. observed no correlation between the two [28]. This complicated situation may have arisen because it is only when soil water content reaches the wilting point of soil organisms (roots or microorganisms) or exceeds the field water holding capacity that it has a significant impact on soil C flux. If the change in moisture does not exceed the upper and lower bounds and is not sufficient to affect soil microbial or root viability, then it will be difficult to clearly measure the effect of moisture on soil C flux because, at this point, the effect of soil moisture can be easily masked by other factors [29].

### Changes in organic carbon content and density of desert soils during BSC succession

In this study, the organic C content and density of desert soils showed regular variations in both horizontal and vertical space. Using BSC succession as the horizontal axis, SOC content and density increased continuously during this process, with the former increasing by 4.89 times and the latter by 3.92 times as the succession progressed from the control to moss sites. This indicates that artificial planting to promote sand fixation can effectively increase the C storage capacity of desert soils, which is consistent with the results of studies on desertification reversal in the Mu Us Desert [30], the vegetation-based sand fixation zone in the Tengger Desert [31], and the vegetation restoration process in the Horqin Desert [32]. First, the succession of BSCs promoted the accumulation of organic carbon in the desert surface soil. The main components of BSCs are cryptogams, such as cyanobacteria, green algae, moss, lichens, etc. These plants can carry out photosynthesis, which is an important channel for CO_2_ to enter the ecosystems present in arid and semi-arid regions. Under suitable light, temperature, and water conditions, the photosynthetic carbon sequestration potential of BSCs mainly depends on their biological composition [33]. Studies have shown that BSCs at the later stages of development contain lichens or mosses and have higher photosynthetic rates than the algae crust, which is the early stage of succession. For example, in the Chihuahuan Desert, photosynthetic rates of BSCs in the later stages of development were 2.4–2.8 times higher than those in the early stage of succession [34]. Thus, the positive succession of BSCs will significantly increase the amount of C entering the ecosystem through BSCs. Second, the synergistic and interactive vegetation-soil feedback relationship is the driving force behind the changes in soil properties. As the population and quantity of surface vegetation increases, the litter and root biomass will accumulate, and a large amount of organic residue will decompose and revert to soil, thus increasing its organic C content [35]. Simultaneously, the surface microhabitat will change under the action of vegetation growth, creating favorable conditions for soil microbial colonization and BSC development. The former’s death, decomposition, and metabolic secretions as well as the latter’s formation of loose, stable humus through cryptogamic cementation, are also direct sources of SOC [36]. Moreover, surface coverage by herbage, shrubs, and BSC can effectively attenuate the activity of mobile dunes and thus reduce the loss of soil C pool due to wind erosion. Using the vertical changes in soil depth as the axis, we found that the organic C content and density of artificially fixed sands decreased with increasing depth. The C content of the surface soil (0–10 cm) was 3.84 times that of deep soil (40–60 cm), thus exhibiting marked nutrient surface accumulation. Veldkamp et al. also showed that the 0–30 cm layer of desert soil accounted for 3.84% of the soil C stock in their study area, which demonstrated significant surface C enrichment of desert soils [8]. This was due to the fact that plant-soil interactions mainly occur at the rhizosphere, and the rhizospheric effect of plants not only provides root C input to the soil, but can also improve soil texture, especially the stability of soil aggregates, thus protecting the existing organic C in the soil [37]. In our study area, approximately 70% of the root biomass of *S. cheilophila* and *A. ordosica* shrubs was concentrated in the 0–30 cm soil layer, and hence the surface soil was inevitably the primary site for nutrient uptake and exudate secretion by the roots to modify soil texture [38]. Atmospheric dust fall and the input of organic residues all occur in the surface and shallow rhizospheric environments, where SOC is mainly accumulated. The study area is located in the semi-arid zone, where precipitation is scarce, water infiltration is difficult, and leaching action is weak. These factors have given rise to difficulties in shifting exogenous organic C to the deep soil, thereby forming a vertical pattern from nutrient-rich to nutrient-poor.

### Synergistic relationship between desert soil surface carbon emission and organic carbon sequestration

**Fig. 5:**
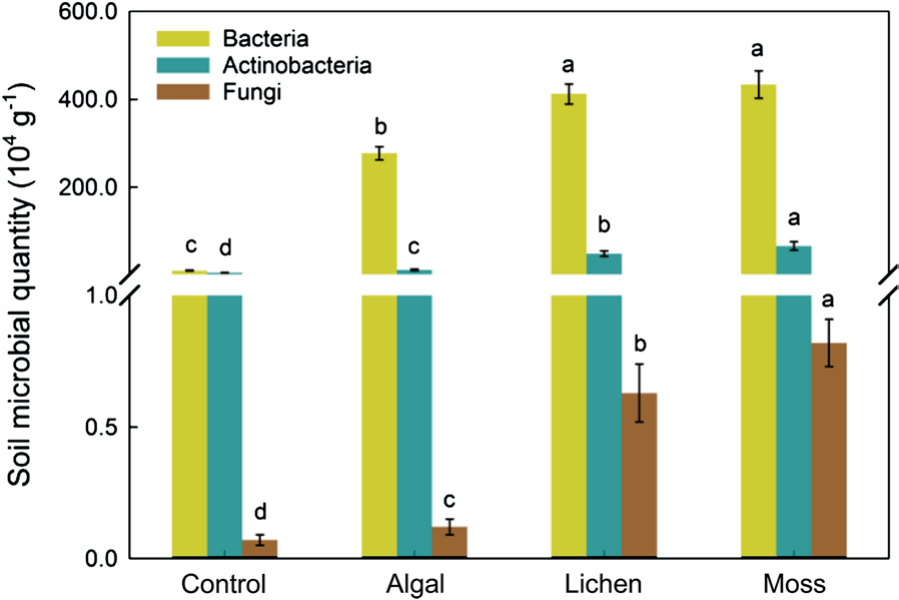
Characteristics of soil microorganisms across sampling sites. Quantitative characteristics of soil microorganisms in the control, algal, lichen, and moss sites. Different letters indicate significant differences between plots. Data are shown as means with standard errors

In this study, the annual C flux at the soil surface increased by 4.08 times from the control to moss sites during the growing season, which was consistent with the analysis performed by Gao et al. [39]. The results showed that the annual soil surface C emissions were positively correlated with SOC density in the 0–10 and 10–20 cm layers. Fan et al. also found that the increase in soil C input was a major contributor to the increase in soil C emissions with the process of vegetation succession [40]. This is mainly because a large component of soil CO_2_ flux is produced by the heterotrophic respiration of microorganisms, and the number of microorganisms determines the level of flux [41]. In this study, the number of microorganisms in the soil under the BSC cover showed a clear trend of significant increase with BSC development (Fig. 5), and this increase was mainly dependent on the continuous supply of soil substrate. SOC is the main C source required for microbial proliferation, and its availability and content can directly affect the quantity and activity of microbial communities. In addition, with the involvement of soil microorganisms or animals, SOC is continuously decomposed and transformed into inorganic C and released as CO_2_ [42]. Moreover, although the desert soil exhibited net absorption of CH_4_ throughout the growing season, the amount of CH_4_ absorbed was very low, and the offset effect on C emissions was not significant. In semi-arid desert areas, artificial vegetation construction can promote rapid restoration of vegetation and the succession of BSCs. During this process, both vegetation and soil can absorb and fix a large amount of C. Although the rate of soil respiration will increase, sand-binding area will act as a huge C sink.

## Conclusion

Following artificial sand fixation by vegetation in a semi-arid desert, the changes in microhabitats gave rise to the formation and succession of BSCs, which led to significant changes in soil C emission patterns. During the growing season, vegetation restoration and BSCs succession can effectively increase CO_2_ emission and the CH_4_ absorption. Furthermore, CO_2_ fluxes were characterized by distinct seasonal dynamics, which was not the case with regard to CH_4_. Soil temperature was observed to be the major factor controlling CO_2_ flux; however, it did not influence CH_4_ absorption in desert soils, and soil water content had a weak effect on both CO_2_ and CH_4_ fluxes. During vegetation restoration and BSCs succession, the organic C content and TOC density of desert soils gradually increased, with clear signs of surface accumulation. The annual soil surface C emissions also showed an increasing trend, which was positively correlated with SOC density. The results of the present study illustrate that vegetation restoration and BSCs succession could increase soil C sequestration in desert soil.

## Materials and methods

### Study area profile

The study area was located in Jungar Banner of Ordos City, Inner Mongolia Autonomous Region, which is a typical desert geomorphological type of eastern Hobq Desert. The study area has a temperate continental climate, characterized by a clear concurrence of precipitation and high temperatures. Its springs and winters are dry and windy, while its summers and autumns are hot with concentrated precipitation. The average annual temperature is 6.1–7.1 °C, the average annual precipitation is 240–360 mm, the average annual evaporation is 2560 mm, the average annual sunshine duration is 3138 h, the average annual frost-free period is 130–140 days, and the average annual wind speed is 3.3 m s^−1^. The soil in the study area was mainly aeolian sandy soil, comprising 2.61% clay and silt (< 0.05 mm), 3.92% ultrafine sand (0.05–0.1 mm), and 92.94% sand (0.1–1 mm). The main plant species included *Salix cheilophila, Caragana korshinskii, Hedysarum mongolicum, Artemisia ordosica, Salsola collina, Psammochloa villosa, and Agriophyllum squarrosum.* After the artificial fixation of sand in the study area, BSCs succession occurred gradually; algae, lichen, and moss crusts constitute the entire BSCs succession sequence.

### Sample sites

The sample sites in the eastern Hobq Desert were divided according to the degree of vegetation restoration and the characteristics of BSCs development into the following types: (1) Algal crust-fixed sand (algal), where *S. cheilophila* cuttings were placed in a grid-like pattern in semi-mobile dunes to form a live biological sand barrier, improve the surface vegetation cover, and promote sand fixation; the vegetation restoration period was 8 years, and black mottled algal crusts had formed on the surface (chlorophyll a content: 0.31 μg g^−1^; scytonemin content: 0.28 μg g^−1^); (2) Lichen crust-fixed sand (lichen), which was afforested with *S. cheilophila* cuttings in bands to form stable *S. cheilophila* communities after pruning; the vegetation restoration period was 18 years, and dark brown patchy lichen crusts had formed on the surface (chlorophyll a content: 0.95 μg g^−1^; scytonemin content: 1.72 μg g^−1^); (3) Moss crust-fixed sand (moss), which was initially afforested with *S. cheilophila* cuttings in bands that eventually formed clusters of “*S. cheilophila* islands” with relatively large crowns through natural succession and had a large number of *A. ordosica* growing in the inter-island open space; the vegetation restoration period was 26 years, and the vegetation coverage was extensive, litter layer was thick, and continuous grayish-green moss crust had formed (chlorophyll a content: 1.93 μg g^−1^; scytonemin content: 7.62 μg g^−1^); (4) Control sample sites (control), which were bare mobile dunes with virtually no vegetation cover, only a few annual herbaceous plants, and strong wind erosion. The basic conditions of the sample sites are listed in Table 5.

**Table 5:** Conditions of different sample sites

Site	Location	Altitude (m)	Vegetation coverage (%)	Dominant species	BSCs thickness (mm)	BSCs coverage (%)	Herbage density (plants m^−2^)	Shrub density (plants·hm^−2^)
Control	110°46′ 33.378″ E, 40°04′ 49.183″ N	1198	2.65 ± 0.18	*Psammochloa villosa* + *Agriophyllum squarrosum*	0	0	15 ± 3	0
Algal	110°47′ 29.805″ E, 40°04′ 34.179″ N	1115	38.63 ± 2.74	*Salix psammophila* + *Hedysarum mongolicum*	1.18 ± 0.06	11.31 ± 1.22	54 ± 6	121 ± 4
Lichen	110°46′ 56.978″ E, 40°04′ 40.868″ N	1147	55.32 ± 4.33	*Salix psammophila* + *Caragana korshinskii*	8.84 ± 1.63	24.66 ± 5.42	134 ± 21	174 ± 7
Moss	110°46′ 28.344″ E, 40°04′ 45.998″ N	1159	65.76 ± 8.12	*Salix psammophila* + *Artemisia ordosica*	13.66 ± 3.14	33.87 ± 4.78	177 ± 32	133 ± 12

*Control* mobile dunes, *Algal* algal crust-fixed sand, *Lichen* lichen crust-fixed sand, *Moss* moss crust-fixed sand, *BSCs* biological soil crust

### Gas sample measurement

Soil CO_2_ and CH_4_ gas samples were collected during the plant growth seasons (May–October) in 2017 and 2018. At each sample site, three 2 m × 2 m gas sampling plots were selected on a relatively flat terrain, and all herbage within the plots was removed to ensure BSCs integrity as much as possible. CO_2_ and CH_4_ collections were performed in a closed static chamber consisting of a cylindrical top chamber (diameter: 320 mm; height: 600 mm) and a base. The top wall of the chamber was equipped with a fan to ensure even mixing of gases in the chamber, and the base was embedded in the soil to a depth of 15 cm. The top chamber was fastened to the base, 2 min prior to each sampling session, and water was injected into the grooves of the base to seal it in order to prevent gas exchange between the inside and outside of the chamber during the sampling process. Sampling was conducted three times per month with intervals of ~ 10 days at all four sample sites. Each sampling time was fixed at 09:00–12:00 am to reduce systematic errors. Timing began when the top chamber was fastened to the base, and gas samples were collected in triplicate (50 mL per sampling bag) at 0, 15, and 30 min. The sampling tool was a medical syringe with a three-way valve, and the gas samples were stored in aluminum foil gas sampling bags.

The gas samples were brought back to the laboratory and stored at a low temperature (− 4℃). The CO_2_ and CH_4_ concentration in the gas samples were measured using a gas chromatograph (Agilent 4890D, USA), and the measurement was completed within 7 days.

### Measurement of soil hydrothermal factors

The meteorological data of the study area were recorded by a small automatic weather station (HOBO, USA). Soil hydrothermal factors were measured dynamically in parallel with gas sampling, involving the stratified measurement of soil temperature and water content at different soil depths of 0–10 cm, 10–20 cm, 20–40 cm, and 40–60 cm using a rapid moisture meter (TRIME PICO, Germany).

### Measurement of SOC and microorganisms

Two soil surveys were conducted at the sample sites in August 2017 and August 2018. Three soil profiles were randomly excavated within each sample site, and after determining the soil horizons, 200 g of mixed samples were obtained at the profile depths of 0–10, 10–20, 20–40, and 40–60 cm, packed into non-woven bags, and brought back to the laboratory. After removing plant roots and gravel, the samples were air-dried naturally, and the organic C content was determined by potassium dichromate-concentrated sulfuric acid oxidation subjected to external heating. The soil bulk density was measured using the volumetric ring method. The volume of the ring was 100 cm^3^, and the determination was repeated three times for each layer. To determine the quantity of soil microbial communities, only soil samples collected from the surface soil (0–10 cm) under crust cover were used, and the number of soil bacteria, actinomycetes, and fungal strains was determined using a quantitative fluorescence polymerase chain reaction assay. UltraClean DNA Isolation Kit (Mo Bio Laboratories, Solana Beach, CA, USA) was used to extract total microbial DNA, a C1000TM Touch Thermal PCR instrument was used for DNA amplification, and the number of microbial strains present in each sample was determined by Fluorescence Ration PCR (Bio-Rad).

### Data processing

Soil CO_2_ and CH_4_ flux was calculated as the amount of gas exchange per unit area of soil based on the changes in gas concentration over time, using the following equation:

$$F = \rho \cdot h \cdot \frac{dC}{{dt}} \cdot \frac{273}{{273 + T}},$$

where *F* is the measured gas flux (CO_2_ unit mmol m^2^ h^−1^; CH_4_ unit µmol m^2^ h^−1^),* ρ* is the gas density (kg m^−3^) under standard conditions, *h* is the static closed chamber height (m), *dC/dt* is the slope of the gas concentration change inside the chamber, and* T* is the average temperature (°C) inside the chamber at the time of sampling.

The annual soil surface C flux (gC m^−2^ year^−1^) during the growing season was calculated using the cumulative method, i.e., the cumulative flux was calculated by multiplying the average measured soil CO_2_ and CH_4_ flux for each month by the number of days in the month as the step size. CH_4_ emissions were converted into CO_2_ emissions equivalents using a factor of 28 [43].

SOC density is the amount of SOC stored per unit area at a given soil depth. The organic C density in layer *i of* the soil profile was calculated using the following equation:

$$SOC_{i} = C_{i} \times D_{i} \times H_{i} \times \left( {1 - G_{i} } \right)/100,$$

where *SOC*_*i*_ is the organic C density of layer *i* (kg·m^−2^), *C*_*i*_ is the organic C content of layer *i* (g·kg^−1^), *D*_*i*_ is the bulk density of layer *i* (g·cm^−3^), *H*_*i*_ is the thickness of layer *i* (cm), and *G*_*i*_ is the gravel volume content of layer *i* (%). The TOC density of the soil profile was obtained by summing up the organic C density of each soil layer.

Data processing and graph plotting were performed using Excel and SigmaPlot 14.0, respectively, and statistical analyses were performed using SPSS 20.0. The least significant difference method was used to test the significance of differences in soil CO_2_ and CH_4_ flux, hydrothermal factors, SOC density, and other indicators among different sample sites (α = 0.05). ANOVA and Pearson's test were used to analyze the correlation between the variables. The data in the tables and figures are presented as mean ± standard error.

## Data Availability

The datasets generated during and/or analyzed during the current study are available from the corresponding author on reasonable request.

## Abbreviations

ANOVA: Analysis of variance
BSC: Biological soil crust
SOC: Soil organic carbon
TOC: Total organic carbon
